# Management of Acute Appendicitis During the COVID-19 Pandemic: A Single-Centre Retrospective Cohort Study

**DOI:** 10.7759/cureus.37193

**Published:** 2023-04-06

**Authors:** Zeeshan Afzal, Ishtiyaq Bukhari, Sumit Kumar, Abdulqudus Deeknah, Winnie Lei, Stefan Mitrasinovic, Onton Chan, Francesca E Francis, Kanagasingham S Satheesan

**Affiliations:** 1 Department of Surgery, Addenbrooke's Hospital, Cambridge University Hospitals NHS (National Health Service) Foundation Trust, Cambridge, GBR; 2 Department of Surgery, Peterborough City Hospital, Peterborough, GBR; 3 Department of Surgery, University of Cambridge, Cambridge, GBR; 4 Milner Therapeutics Institute, University of Cambridge, Cambridge, GBR; 5 Leicester Medical School, University of Leicester, Leicester, GBR

**Keywords:** conservative and surgical treatment, open and laparoscopic surgery, acute appendicitis, pandemic, covid-19

## Abstract

Background: During the coronavirus disease 2019 (COVID-19) pandemic, the management of acute appendicitis shifted towards non-operative management in the United Kingdom (UK). The open approach was recommended over the laparoscopic approach due to the risk of aerosol generation and subsequent contamination. The aim of this study was to compare the overall management and surgical outcomes of the patients treated for acute appendicitis before and during the COVID-19 pandemic.

Materials and Methods: We performed a retrospective cohort study at a single district general hospital in the UK. We compared the management and outcome of the patients diagnosed with acute appendicitis before the pandemic, from March to August 2019, and during the pandemic, from March to August 2020. We looked at the patient demographics, methods of diagnosis, management, and surgical outcomes for these patients. The primary outcome of the study was the 30-day readmission rate. Secondary outcomes included length of stay and post-operative complications.

Results: Over the period of six months, a total of 179 patients were diagnosed with acute appendicitis in 2019 (Pre-COVID-19 pandemic, from March 1, 2019, to August 31, 2019) versus 152 in 2020 (during the COVID-19 pandemic, from March 1, 2020, to August 31, 2020). For the 2019 cohort, the mean age of the patients was 33 (range 6-86 years), 52% (n=93) were female, and the mean BMI was 26 (range 14-58). For the 2020 cohort, the mean age was 37 (range 4-93 years), 48% (n=73) of the patients were female, and the mean BMI was 27 (range 16-53). At the first presentation, in 2019, 97.2% of the patients (174 out of 179) received surgical treatment compared to 70.4% (107 out of 152) in 2020. Three per cent of the patients (n=5) were managed conservatively in 2019 (two out these failed conservative management) as compared to 29.6% (n=45) in 2020 (21 of these failed conservative management). Pre-pandemic, only 32.4% (n= 57, ultrasound (US) scan: 11, computer tomography (CT) scan): 45, both US and CT: 1) of the patients received imaging to confirm the diagnosis as compared to 53.3% during pandemic (n=81, US scan: 12, CT scan: 63, both US and CT: 6). Overall, the CT to US ratio increased. We found that during 2019, 91.5% (n=161/176) of the patients who received surgical treatment went through laparoscopic surgery as compared to only 74.2% (n=95/128) in 2020 (p<0.0001). Postoperative complications occurred in 5.1% (n=9/176) of the surgical patients in 2019 as compared to 12.5% (n=16/128) in 2020 (p<0.033). The mean length of hospital stay in 2019 was 2.9 days (range 1-11) versus 4.5 days in 2020 (range 1-57) (p<0.0001). The 30-day readmission rate was 4.5% (8/179) versus 19.1% (29/152) (p<0.0001). The 90-day mortality rate was zero for both cohorts.

Conclusion: Our study shows that the management of acute appendicitis changed due to the COVID-19 pandemic. More patients went through imaging, especially CT scans for diagnosis and received non-operative management with antibiotics only. The open surgical approach became more common during the pandemic. This was associated with longer lengths of hospital stay, more readmissions, and an increase in postoperative complications.

## Introduction

Acute appendicitis, described as acute inflammation of the vermiform appendix [1], is one of the most common causes of abdominal pain in both adults and children [2]. The commonest aetiology is thought to be luminal obstruction of the appendix [3,4]. Its incidence is reported as 5.7-50 patients per 100,000 inhabitants per year [2]. It is a very common surgical emergency encountered worldwide [1,5], leading to around 50,000 appendectomies performed annually in the United Kingdom (UK) alone [1].

The management of acute appendicitis includes the use of antibiotics and operative management (often both) but this still, to date, presents a dilemma for a clinician [6]. Although the antibiotic-alone strategy can avoid complications from general anaesthesia and surgery itself, it has a recurrence rate of up to 39% after five years [2].

However, during the coronavirus disease 2019 (COVID-19) pandemic, the management of acute appendicitis shifted predominantly towards non-operative management in the UK for the very first time. An open approach was recommended over a minimally invasive approach due to the risk of aerosol generation and subsequent contamination [7].

The aim of this study was to compare the overall management and surgical outcomes of the patients treated for acute appendicitis before and during the pandemic. The primary outcome of the study was the 30-day readmission rate. Secondary outcomes included length of stay and post-operative complications.

## Materials and methods

Study design and population

We performed a retrospective cohort study at a single district general hospital, Peterborough City Hospital, North West Anglia NHS Foundation Trust, Peterborough, United Kingdom, between March 2019 and August 2020. The study compared the management and outcome of patients with acute appendicitis on the index admission before and during the COVID-19 Pandemic. The patients were divided into two main groups: The pre-Pandemic group from March 1, 2019, to August 31, 2019, and the Pandemic group from March 1, 2020, to August 31, 2020. Patients were identified using the hospital’s coding system and cross-checked with the hospital’s electronic theatre operating system. All the patients (age greater or equal to 2 years) with a clinical or radiological diagnosis of appendicitis were included in the study during the defined time periods. This study was formally registered and accepted at the Quality Governance and Compliance Department, North West Anglia NHS Foundation Trust, United Kingdom (Audit number 3438).

Data collection

Data for both groups were collected using electronic patient notes. This included patient demographics including age, gender, body mass index (BMI), diagnostic criteria for the diagnosis of acute appendicitis (clinical vs. radiological), management pathways (conservative vs. surgical), and radiology imaging reports. We also recorded the patient outcomes including histology results for surgical patients, length of hospital stay (LOS) for the index admission, recurrence of disease, surgical complications (including ileus, intra-abdominal collections, respiratory tract infections such as hospital-acquired pneumonia (HAP), surgical site infections (SSIs), readmission within 30 days, and mortality within 90 days.

Clinical diagnosis was based on the presentation of abdominal pain with localised tenderness and rebound/percussion tenderness in the right iliac fossa (RIF) with associated anorexia and nausea and/or vomiting. Radiological diagnosis was based on the CT findings of thickened appendix wall with/without a faecolith at the base, with surrounding fat stranding and/or peri-appendiceal fluid.

Conservative management was defined as a non-operative approach with the use of antibiotics only at initial presentation not requiring any surgical procedure during the index admission. Surgical management was defined as an operative approach at the initial presentation with or without the use of antibiotics. Conversion from a laparoscopic to an open approach was defined as an additional procedure performed via an incision at the right iliac fossa or midline laparotomy. Failure of conservative management was defined as worsening or non-resolution of clinical signs, symptoms, and/or biochemical markers including white cell count (WCC) and C-reactive protein (CRP).

Post-operative ileus was defined as the symptomatic absence of bowel function for more than 72 hours after surgery requiring the insertion of a nasogastric tube (NGT). SSI was defined by the clinical manifestation of inflammation including pain, erythema, and discharge, regardless of the microbiological culture results. Respiratory tract infection was diagnosed on the bases of positive radiological findings on either a plain chest X-ray film or computer tomography (CT) scan reports. Intra-abdominal collection was defined as the presence of any radiological evidence of organised and/or walled-off fluid in the abdomen after the surgery.

Statistical analysis

Descriptive data were reported as mean (with either standard deviation (SD) or range) or number/total (%) as appropriate. When comparing nominal data, Chi-squared or Fisher’s exact test was used. For continuous, not normally distributed data, the Mann-Whitney U test was used. A p-value <0.05 was regarded as the level of statistical significance. Data analysis was performed using R (version 4.2.2; R Foundation for Statistical Computing, Vienna, Austria).

## Results

A total of 179 patients were diagnosed with acute appendicitis in 2019 (Pre-COVID-19 pandemic, from March 1, 2019, to August 31, 2019) versus 152 in 2020 (during COVID-19 Pandemic, from March 1, 2020, to August 31, 2020). For the Pre-Pandemic group, the mean age of the patients was 33 (range 6-86 years), 52% (n=93) were female, and the mean BMI was 26 (range 14-58). For the Pandemic group, the mean age was 37 (range 4-93 years), 48% (n=73) of the patients were female, and the mean BMI was 27 (range 16-53) (Table 1).

**Table 1:** Patient Demographics

		Pre-Pandemic group (n=179)	Pandemic group (n=152)
Age	Mean (Range)	33 (6-86)	37 (4-93)
Gender	Male	86 (48%)	79 (52%)
	Female	93 (52%)	73 (48%)
BMI	Mean (Range)	26 (14-58)	27 (16-53)

At the first presentation in the Pre-Pandemic group, 97.2% of the patients (174 out of 179) received surgical treatment compared to 70.4% (107 out of 152) in the Pandemic group. Three per cent of the patients (n=5) were managed conservatively in the Pre-Pandemic group (of which two failed conservative management) as compared to 29.6% (n=45) in the Pandemic group (of which 21 failed conservative management). In the Pre-Pandemic group, only 32% (n= 57, US scan: 11, CT scan: 45, both: 1) of the patients received imaging to confirm the diagnosis as compared to 53% in the Pandemic group (n=81, US scan: 12, CT scan: 63, both: 6). Overall, the CT to US ratio increased.

In the Pre-Pandemic group, 91.5% (n=161/176) of the patients who received surgical treatment went through laparoscopic surgery (vs. open surgery) as compared to only 74.2% (n=95/128) in the Pandemic group (p<0.0001). When we analysed the histology data, we found that in the Pre-Pandemic group, 77% (135/176) of patients who were operated on had histology-proven acute appendicitis, 1% (n=1) of patients were diagnosed with carcinoid tumour, 1% (n=1) with inflammatory bowel disease, and 21 % (n=35) had normal appendix. On the other hand, in the Pandemic group, 84% (108/128) were diagnosed with histology-proven acute appendicitis and only 16% (n=20) had normal appendix on histology.

The primary endpoint of the study, the 30-day readmission rate, was 4.5% (8/179) in the Pre-Pandemic group versus 19.1% (29/152) in the Pandemic group. This was statistically significant (p <0.0001).

The post-operative complication rate was a secondary endpoint of the study. In the Pre-Pandemic group, 5.1% (n=9/176) of the surgical patients developed post-operative complications: Ileus (n=6), collections (n=1), and SSI (n=2). On the other hand, in the Pandemic group, 12.5% (n=16/128) developed postoperative complications: Ileus (n=6), collections (n=4), HAP (n=3), SSI (n=3) (p <0.0001). Another secondary endpoint of the study was the mean LOS, which in the Pre-Pandemic group was 2.9 days (range 1-11) versus 4.5 in the Pandemic group (range 1-35). This was statistically significant (p <0.0001). The 90-day mortality rate was zero for both cohorts (Table 2).

**Table 2:** Results *Chi-squared test, †Fisher’s exact test, ‡Mann-Whitney test, ±Mean

		Pre-Pandemic group (n=179)	Pandemic group (n=152)	p-value
Treatment				< 0.00001*
	Surgical	174 (97.2%)	107 (70.4%)	< 0.00001†
	Conservative	5 (2.8%)	45 (29.6%)	< 0.00001†
	Failed conservative	2/5 (40.0%)	21/45 (46.7%)	1†
Imaging				1†
	Imaging done (vs. no imaging)	57 (32.4%)	81 (53.3%)	0.0001†
	Ultrasound scan only	11 (6.1%)	12 (7.9%)	0.496†
	CT scan only	45 (25.1%)	63 (41.4%)	1†
	Both Us and CT scans	1 (0.6%)	6 (3.9%)	0.239†
Operation				0.000138*
	Laparoscopic surgery	161/176 (91.5%)	95/128 (74.2%)	0.0001†
	Open surgery	10/176 (5.7%)	27/128 (21.1%)	0.0001†
	Converted (From laparoscpy to open)	5/179 (2.8%)	6/128 (4.7%)	0.536†
Histology				
	Normal	35/176 (19.9%)	20/128 (15.6%)	0.3685†
	Appendicitis	139/176 (79.0%)	108/128 (84.4%)	0.2975†
	Malignancy	1/176 (0.6%)	0/128 (0%)	1
Complications				
	Total	9/176 (5.1%)	16/128 (12.5%)	0.033†
	Ileus	6/9 (66.7%)	6/16 (37.5%)	0.226†
	Collections	1/9 (11.1%)	4/16 (25.0%)	0.621†
	Hospital acquired Pneumonia	0/9 (0%)	3/16 (18.8%)	0.28†
	Surgical site infection	2/9 (22.2)	3/16 (18.8%)	1†
Length of stay±		2.88 (SD 2.40, range 1-11)	4.53 (SD: 5.79, range 1-57)	< 0.0001 ‡
30-day readmission ±		8 (4.5%)	29 (19.1%)	<0.0001†
90-day mortality±		0 (0%)	0 (0%)	

## Discussion

The management of acute appendicitis varies throughout the different healthcare setups due to local guidelines, which are mostly based on the availability of financial and other resources including overnight radiology/imaging such as ultrasound and/or CT scans, laparoscopic surgical expertise, etc. Although surgical intervention is considered the gold standard choice of treatment for acute appendicitis, there is evidence eluding towards the benefits of conservative management mitigating post-operative complications and re-operation rates [8-11].

The COVID-19 pandemic presented a new set of concerns including the spread of deadly viral infection, lack of availability of personal protective equipment at the hospitals, and acute shortage of healthcare staff. These challenges had a huge impact on patient care and management not only in primary care but also in hospital settings. Surgical procedures, predominantly laparoscopic, are considered aerosol-generating procedures (AGPs); therefore, the risk of infection spread is greater and more concerning [12]. Since there was a lack of clear evidence at the time of the pandemic and a growing concerns over the spread of infection with AGPs, it was recommended to change the surgical practice including delaying surgical procedures and considering alternative management strategies, if possible [13,14].

Due to the reasons mentioned above and uncertainty towards peri-operative outcomes due to COVID-19 exposure, the paradigm of the management of acute appendicitis also moved more towards conservative management with antibiotics only. The open surgical approach was preferred to laparoscopic procedures as an attempt to reduce the risk of infection spread [15].

The result of our study demonstrates this paradigm shift at our local hospital. We found a statistically significant difference in the choice of management of acute appendicitis before and during the COVID-19 era. As compared to the pre-pandemic times, during the pandemic more patients were treated conservatively with antibiotics only. This move towards the trial of conservative management by choice produced a cohort of patients that initially did not respond to the antibiotics-only treatment and had to be operated on later in the course of illness. The choice of surgical approach used for the management of acute appendicitis before and during the COVID-19 pandemic also showed a statistically significant difference; the open surgical approach was more common during the COVID-19 pandemic.

Although CT imaging is highly sensitive and specific for the diagnosis of acute appendicitis, it is associated with radiation risk and therefore US imaging can aid in diagnosing acute appendicitis or ruling out other differential diagnoses [16-18]. From our study results, we also observed that the use of radiological imaging increased significantly during the pandemic. In particular, the use of CT imaging increased as compared to the US imaging modality. This helped with establishing immediate diagnosis more accurately, aiding towards selecting the management pathway for the patients appropriately in the early stages of hospital admission and avoiding unnecessary invasive surgical procedures. 

However, an increase in failed conservative management and open surgical procedures contributed towards higher post-operative complications such as collections, pneumonia, and surgical site infections. This is ultimately reflected in the longer hospital stays and more readmissions during the pandemic. The patients who developed post-operative pneumonia all tested negative for COVID-19 infection; however, this could easily be categorised as an anaesthetic complication as well. Our primary (30-day hospital re-admission) and secondary endpoints (LOS and post-operative complications) showed statistically significant differences.

The main limitations of the study are that it’s a retrospective study done at a single centre with small cohort size. The time duration of the study could also have been improved by comparing patients for 12 months instead of six months. Similarly, the follow-up period is only up to 90 days, and if extended over three to five years will theoretically highlight a group of patients with recurrence of appendicitis due to conservative management with antibiotics only. This can allow us to perform long-term analysis on the success of the chosen management strategy, the recurrence rate of the disease, and other surgical complications such as wound infections, incisional hernias, etc.

Nonetheless, this is a pertinent study which highlights the change in clinical decision-making in the management strategy for acute surgical pathology during the COVID-19 pandemic.

## Conclusions

Our study shows that the management of acute appendicitis changed due to the COVID-19 pandemic. It also highlights the precautionary measures taken to protect the medical staff. More patients went through imaging especially CT scans for diagnosis and received non-operative management with antibiotics only. The open surgical approach became more common during the pandemic as compared to the laparoscopic approach. This contributed towards longer LOS, more readmissions, and an increase in post-operative complications.
